# Case of Hysterical Knee

**Published:** 1894-10-20

**Authors:** A. G. Miller

**Affiliations:** Lecturer on Clinical Surgery and Surgeon to the Royal Infirmary, Edinburgh


### CASE OF HYSTERICAL KNEE.

By A. G. Miller, M.D., F.R.C.S.E., Lecturer on Clinical Surgery and Surgeon to the Royal Infirmary, Edinburgh.

In cases of so-called hysterical joint one of the most important and most difficult points is the diagnosis. The difficulty often arises from the reluctance one has to say that the patient is humbugging, for, whenever one's mind is made up, the diagnosis is then clear and undeniable. Of course there is often difficulty in other cases of joint affection, but then it is between one diseased condition and another. In the present case the diagnosis is between a diseased and a simulated condition, between a serious affection and nothing.

By my last remark I do not mean to say that a hysterical affection is nothing. I know that these affections are real enough to the patient, and that there is sometimes a very great difficulty in drawing the line between so-called hysteria and actual disease. What I mean is that in *joint* affections the diagnosis is between something and nothing, because in a hysterical joint there is really nothing wrong with the joint. The joint in all its constituent parts is healthy. The diseased or deranged condition is in the nervous system. This may be proved in two ways. In the first place the joint can be made out to be perfectly healthy by manipulation, if need be, with the assistance of anæsthesia. In the second place, the patient can use the joint perfectly if convinced that it is healthy, or when not observed.

I have met with hysterical joints in young women, middle-aged women, and in boys. Two cases of hysterical hip occurred in middle-aged ladies, who got well with change of air, tonics, and massage. Two cases of apparent hip disease in boys were completely cured by circumcision. Two cases of hysterical knee occurred in young women, one of which has been a long time under my observation, and, as it is a very remarkable case, I venture to publish it.*

M. M., female, æt. 22, single, house servant, admitted to the Edinburgh Royal Infirmary under Mr. Miller's care (for the first time), February, 1889. She was in hospital from February to June, 1889; for three weeks in October, 1889; for three weeks in March, 1890; for one week in May, 1890; from July to November, 1890; for six weeks in November and December, 1891; from November, 1892, to February, 1893; and again for a month in November and December, 1893. *Complaint*—Pain in left knee. *Physiognomy*—Patient is stout and healthy-looking, fair-complexioned, and well nourished. *History* (taken 1889)—Had gastric fever when seven years old. Seven years ago suffered from housemaid's knee. About a year ago was operated on by Professor Annandale for wry-neck, which had existed for about a year. (There is no trace of wry-neck or of an incision.) About three years ago received a kick on left knee from a boy, but this did not cause her much trouble till about a year after, when she became unable to kneel on account of pain. Gradually she became lame, and had to give up work. She was admitted to the Chalmers' Hospital some months ago, when blistering and rubbing were tried. Dr. Balfour considered the affection to be hysterical.

*Family History.*—Mother died of heart disease, æt. 44. Father living, and healthy. Four sisters and two brothers, alive and healthy. Four brothers died in infancy.

*Condition of Left Knee.*—Neighbourhood of joint swollen, limb rigid (sometimes straight and sometimes flexed); pain on pressure at somewhat varying points; hyper-sensitiveness all round the joint; patient cries out on least touch; thigh somewhat smaller than right side; muscles hard and rigidly contracted; patella fixed (evidently by muscular contraction); synovial membrane cannot be made out as being thickened or otherwise diseased; no enlarged veins, or long silky hairs over joint; joint, though enlarged, has not lost characteristic osseous points, and is not ovoid,

* In recording this case I quote from the ward reports. As these are very numerous and extensive, it would make the history of the case unnecessarily long and complicated were I to give them fully; I am therefore giving a "digest" of the case.

plenty of subcutaneous fat; leg below the knee swollen, sometimes distinctly œdematous. Patient cries out and resists when any attempt is made to move the limb. On one occasion when patient presented herself at the hospital the limb was drawn up and shortened as in hip disease, and there was a marked lateral curvature of the spine, all of which disappeared when patient was stretched out in bed.

All symptoms disappear under full anæsthesia, and return when patient recovers perfect consciousness.

All organs and functions healthy.

Temperature normal.

Treatment employed at various times—Acupuncture, blisters, massage, Corrigan's button, actual cautery, splints, exploratory incisions and hypnotism.

Comparative measurements of healthy and affected limbs, taken carefully at same level on both limbs:—

| | | | At Patella. | Above. | Below. |
|---|---|---|---|---|---|
| Oct., | 1889. | L. affected side | ... 17 in. | ... 17½ in. | ... 16 in. |
| ,, | ,, | R. healthy side | ... 15½ in. | ... 18 in. | ... 14 in. |
| Nov., | 1891. | L. affected side | ... 16½ in. | ... 16 in. | ... 15½ in. |
| ,, | ,, | R. healthy side | ... 14¾ in. | ... 15 in. | ... 14 in. |
| Dec., | 1891. | L. affected side | ... 15 in. | ... 18 in. | ... 13¼ in. |
| ,, | ,, | R. healthy side | ... 14½ in. | ... 18 in | ... 12½ in. |

On one occasion measurements were made before and during the administration of chloroform (of the affected limb): Before chloroform, patella, 14¾ in.; ankle, 8¼ in.; thigh, 16½ in. Under chloroform, patella, 14½ in; ankle, 8 in.; thigh, 17 in.

The limb was visibly more natural and like the other one when the patient was quite unconscious. Movement was perfectly free. So long as patient had any consciousness she cried out at the slightest touch in the neighbourhood of the joint, and resisted powerfully any attempt to move the joint. Rigidity began to return to the joint long before the patient was conscious, and the joint was practically as before when the patient recovered her consciousness.

The patient always had the curious suspicious expression that is usual in hysterical subjects. Her ovaries never presented anything characteristic of disease.

In this case the difficulty lay, in regard to diagnosis, not in saying what was not wrong with the knee, but what was wrong with it. The actual condition of the joint most resembled white swelling or strumous disease.

The symptoms which resembled a tubercular joint were, enlargement of the joint without discoloration, fixation of the joint by muscular spasm, pain on pressure at certain points and pain on movement, atrophy of the limb above, and œdema of the limb below the joint, history of a slight injury, long continuance of chronicity, and failure of ordinary treatment to benefit the condition. On the other hand, the enlargement of the joint was not typical of tubercle. The swelling was not smooth and ovoid, there were no large veins, or long silky hairs covering the joint, and there was no decided increase of temperature, either locally or generally. Again, the pain on pressure was at uncertain and varying points, as we proved by marking with ink, and the patient's behaviour, crying out and resisting vigorously every attempt to move the joint, was greatly in excess of what one finds in those suffering from chronic tubercular disease. The atrophy of the limb above, and œdema below, the affected joint, were the conditions which were the most difficult to discount. Charcot, however, has proved that these conditions can be simulated in hysterical affections. Lastly, the history of the case was not unlike white swelling. An injury, not very severe; gradual and insidious development of the symptoms; obstinate refusal of the symptoms to give way to ordinary simple remedies. But, on the other hand, the interval between the injury and the commencement of the symptoms (a whole year) was most unusually long, and the resistance of the symptoms to treatment was unnaturally obstinate, and the period during which they had persisted was so long that in a truly tubercular case either cure or utter destruction of the joint would have supervened.

It is evident then that this case, though like a tubercular one, was not characteristic. It had not the true ring. It was not typical in any respect. This of course raised the suspicion of hysteria. Indeed, it is the special characteristic of such hysterical affections that they resemble true diseased conditions, but only resemble them. They are like the disease, but on careful examination the essential characteristics of the simulated disease are awanting. The general features may be wonderfully simulated; the outward appearances may be closely and graphically represented, but the real thing is awanting. Sometimes the true disease is so closely mimicked that it is very difficult to come to an exact diagnosis. Often one is misled, for a time, into treating the hysterical condition as if it were real. And it is better and safer to do so, though it may ultimately be humbling to our professional pride, than to risk ignoring a genuine diseased condition. In our present case, however, we had very positive as well as negative conditions to guide and confirm our diagnosis. In the first place the patient was stout and healthy. To look at she was robust, and she had a good layer of fat all over her, not excepting the affected limb and joint. Indeed, on careful examination, the swelling round the knee, and below it, could be made out to be largely due to a substantial deposit of fat. This of course was against tubercle; for tubercular patients are not generally fat, though I have met with some who were fairly fat, but not in the neighbourhood of the diseased joint. Waste is the characteristic symptom of tubercle. Next the hypersensitiveness of the patient, and the frequent variation of the most painful spot at the joint, were quite characteristic and diagnostic.

One of the special characteristics of a chronic tubercular joint affection is its comparative painlessness. Then there is usually one spot at which there is special pain, aggravated by pressure. It may be over one of the condyles, or over the head of the tibia, but it is fixed and always the same. In the present case the painful spot, though usually somewhere about the head of the tibia where the kick had been, varied considerably from time to time. This was proved in the usual way by making the patient shut her eyes and then marking with ink the spot at which she said she had the greatest pain. As this was repeated from time to time a considerable variance of the spot complained of was noted. Again, although the limb was rigid, splinted by the muscles, as if to prevent pain or movement, the muscular spasm was not constant, was partly voluntary, and the position of the limb varied—it was sometimes flexed and sometimes straight. Now in a tubercular affection of the knee flexion always occurs sooner or later with displacement of the tibia backwards, accompanied and perhaps caused by spastic contraction of the hamstring muscles and atrophy of the extensors.

But I must not prolong my remarks on this case, although I am tempted, seeing it is a most striking example, and there are many points in the history worth dwelling upon. Let me now refer to the main thing on which I founded and maintained my diagnosis, viz., the complete disappearance of all symptoms when the patient was fully under the influence of chloroform. Let me discribe what happened.

As the patient gradually came under the influence of the anæsthetic I began to handle the affected limb. Immediately the patient cried out, shrank from touch. and powerfully resisted all attempts to move the joint, Then, as the reflexes began to be abolished, crying out ceased, and some movement of the joint was permitted, then suddenly, when complete anæsthesia was reached, the limb became perfectly passive in my hands. I could flex perfectly and extend without the least resistance, and the movements of the joint were absolutely natural. Moreover, when the limb was stretched out beside the other, and the two compared together, hardly any difference could be made out by the eye or the hand, and measurement demonstrated that the circumference of the affected joint had become less, while the measurement of the thigh was slightly greater, and of the ankle (which was apparently œdematious) less. Then there happened a remarkable thing. As the patient gradually emerged from under the influence of the anæsthethic the limb gradually stiffened, and in a short time the joint was just as it had been, or nearly so. The muscular spasm and hypersensitiveness began to return long before the patient was perfectly conscious. This shows that, in this patient's case any way, the symptoms of hysteria were not so voluntary and under the control of the patient as we are apt to suppose. Perhaps an acquired habit has something to say in the matter. We all know that a "habit o' swearin'" sometimes asserts itself when the patient is under the influence of chloroform, and that the "alcoholic habit" always exerts a manifest influence on the manner in which the patient takes the chloroform.

There remains but one point on which I need remark—treatment. In this case treatment has failed to do any material good, and this is one of the proofs of the condition being hysterical. If there were any real structural changes in the joint the affection would have been, as I have already said, cured, or would have progressed to the utter destruction of the joint long ago. The patient has over and over again been discharged from the hospital much improved, but has always returned, and is at present much the same as she was in 1889.

Why has all treatment, while always so far benefiting, failed to bring about a cure? Because the patient is still under the belief that there is really something wrong with her knee. On one occasion, when the girl was half under the chloroform, I found that I could make her voluntarily flex and extend her limb by ordering her to do so. This suggested to me that hypnotism might cure her. This was tried by an experienced hypnotist, but he failed utterly in producing hypnotism. The girl would not be hypnotised—she evidently had the stronger will of the two.

It may be asked why I received this patient into my ward so often, and employed so many methods of treatment—going the length of exploratory incisions. I did so in the first place because she was recommended to my care by several physicians as suffering from white swelling, and because some of my surgical colleagues even believed that there was something wrong somewhere. I had doubts myself at first until I had the patient under chloroform. In the second place the patient herself evidently thoroughly believed that her knee joint was diseased, and she evidently suffered pain either locally or mentally. In the third place her case was most instructive and valuable for teaching purposes; and lastly, I was always in hopes that I might find out some way of curing the hysteria. In this I have failed, for the girl still haunts the hospital, as I find from my nurses, but does not like to present herself before the students, because she knows well enough the view we take of her case. She was last seen a month or two ago still limping and complaining of her knee, but managing to walk with it fairly well for all that. She looked well and strong.

